# Vertical transmission of maternal COVID-19 antibodies after CoronaVac vaccine: A case report

**DOI:** 10.1590/0037-8682-0385-2021

**Published:** 2021-11-12

**Authors:** Bruno Thizon Menegali, Fabiana Schuelter-Trevisol, Alexandre Naime Barbosa, Talita Menegali Izidoro, Otto Henrique May Feurschuette, Chaiana Esmeraldino Mendes Marcon, Daisson José Trevisol

**Affiliations:** 1 Universidade do Sul de Santa Catarina, Programa de Pós-Graduação em Ciências da Saúde, Tubarão, SC, Brasil.; 2 Universidade do Sul de Santa Catarina, Faculdade de Medicina, Tubarão, SC, Brasil.; 3 Universidade do Estado de São Paulo, Departamento de Doenças Infecciosas, Botucatu, SP, Brasil.

**Keywords:** Coronavirus infections, Immunization passive, Vaccine

## Abstract

The use of coronavirus disease 2019 RNA vaccines in pregnant women led to reports on the first cases of newborns with antibodies to sudden acute respiratory syndrome coronavirus 2 (SARS-CoV-2), a phenomenon that was unknown when using immunizations with inactivated viruses. Thus, this study aimed to report a case of passive anti-SARS-CoV-2 immunity in a newborn through immunoprophylaxis of a pregnant woman who received the CoronaVac® vaccine in the third trimester of pregnancy. Twenty-four hours after delivery, samples were collected from the newborn and screened by enzyme immunoassays, which revealed antibodies to SARS-CoV-2.

## INTRODUCTION

The novel coronavirus disease (COVID-19) is an extremely contagious respiratory transmissible disease caused by severe acute respiratory syndrome coronavirus 2 (SARS-CoV-2), which in adults may lead to severe viral pneumonia requiring hospitalization in about 10-15% of infected patients and a general lethality rate of 2-3%^1^. Pregnant and postpartum women are more vulnerable to severe COVID-19 infections than their non-pregnant counterparts with the same baseline characteristics^1^ ^,^ ^2^.

The effects of COVID-19 on pregnant women and their babies are still being studied. Pregnancy alters various functions of the human body, leaving women more vulnerable to infectious diseases, including SARS-CoV-2 and its vertical transmission to the fetus; the latter has not yet been ruled out^3^.

SARS-CoV-2 infections present a very heterogeneous clinical scenario that may depend on the viral load and vulnerability of an infected person. Symptoms may include cough, runny nose, fever, sore throat, and dyspnea that can vary from asymptomatic to severe respiratory failure^4^. Although elderly people and adults with comorbidities are the most vulnerable group for aggravation and death, pregnant women and infants aged 0-1 year are also vulnerable to COVID-19 complications^2^ ^,^ ^5^.

There are limited data regarding the safety of vaccines against COVID-19 during pregnancy. Clinical trials on their efficacy and safety did not include pregnant women; therefore, the decision to vaccinate pregnant women against COVID-19 is based on a risk-benefit ratio. In Brazil, pregnant healthcare professionals working on the frontline of the COVID-19 outbreak were immunized with CoronaVac® because this vaccine uses a known and safe technology for pregnancy as long as its use is recommended by a woman’s obstetrician^6^.

This study aimed to report a case of passive transmission of anti-SARS-CoV-2 antibodies through immunoprophylaxis in pregnant women during the third trimester of pregnancy.

## CASE REPORT

T.M.I, a 33-year-old physician, was multiparous. On February 23, 2021, at 34 weeks of gestation, she received the first 0.5 mL dose of the CoronaVac® vaccine (Instituto Butantan, São Paulo, Brazil) containing 600 SU of the inactivated virus antigen to SARS-CoV-2. A second dose of equal volume and composition was administered on March 15, 2021, when she was at 37 weeks of gestation. No complications were detected during prenatal care. She attended 10 antenatal consultations without any symptoms of SARS-CoV-2 infection. There was a weight gain of 14 kg, and she ended the pregnancy at a weight of 110 kg and height of 166 cm.

The delivery took place at 39 weeks of gestation by cesarean section on April 9, 2021. The newborn was male, weighed 3.44 kg, was 48 cm long, and had a head circumference of 33 cm. He was breastfed, and a comprehensive physical assessment revealed that he was healthy. The Apgar scores at 1 and 5 minutes were 9 and 10, respectively.

Blood specimens were collected by peripheral venipuncture 24 h after birth to detect neutralizing antibodies against SARS-CoV-2/COVID-19. The serological test, carried out by enzymatic immunoassay (cPass™ SARS-CoV-2 Neutralization Antibody Detection Kit, GenScript, Make Research Easy), showed a result of 22%, which was considered positive based on the cutoff value of 20%. The cPass™ SARS-CoV-2 Neutralization Antibody Detection Kit is a blocking enzyme-linked immunosorbent assay (ELISA) intended for the qualitative direct detection of total neutralizing antibodies to SARS-CoV-2 in human serum and K2-EDTA plasma as a detection tool. Using purified receptor binding domain, protein from the viral spike (S) protein, and the host cell receptor ACE2, this test is designed to mimic the virus-host interaction by a direct protein-protein interaction in a test tube or a well of an ELISA plate. This highly specific interaction can then be neutralized in the same manner as in a conventional virus neutralization test. 

This study was approved by the Research Ethics Committee of the University of Southern Santa Catarina (opinion no. 4.728.687) on May 24, 2021. Informed consent was obtained from the mother of the child prior to the data collection. 

## DISCUSSION

The inactivated SARS-CoV-2 vaccine with aluminum hydroxide developed by Sinovac Life Sciences Co. Ltd., known as CoronaVac®, has been shown to be safe and effective for inducing neutralizing specific antibodies^7^.

The Butantan Institute (Brazil) conducted a study of 9,823 participants who received two doses of CoronaVac between July and December 2020. The primary efficacy rate was 50.7% (95% confidence interval [CI], 36.0-62.0) against symptomatic COVID-19, while the secondary efficacy was 83.7% (95% CI, 58.0-93.7) against moderate cases requiring assistance and 100% (95% CI, 56.4-100.0) against severe cases^7^.

In this case report, the CoronaVac vaccine was administered to a pregnant woman and assumed to be safe according to the literature because inactivated vaccines consist of dead antigens and are safe for this specific group without any major adverse effects or damage to the fetus. Studies have revealed that the vaccination of pregnant women against influenza, hepatitis A, and tetanus, for example, is common practice^6^ ^,^ ^8^.

Studies comparing the behavior of COVID-19 between pregnant and non-pregnant women have been conducted to enhance antenatal care and find safe alternatives to immunization during pregnancy. These studies showed that pregnant women who acquired SARS-CoV-2 and developed infectious symptoms were at great risk of hospital admission; the development of severe conditions requiring intensive care unit admission; progression to respiratory failure requiring invasive ventilation (endotracheal intubation); and, consequently, a high mortality risk. From an obstetric point of view, high rates of preterm births and the prevalence of operative deliveries have been observed as well^2^ ^,^ ^5^.

In this case report, passive immunity may have occurred via the transplacental route measured by total antibodies after exposure to inactivated antigens of the vaccine administered in the third gestational trimester. Immunoglobulin G transfer from the mother to the fetus begins at the end of the first trimester of gestation and increases throughout pregnancy, ranging from 10% of maternal concentration in weeks 17-22 to 50% in weeks 28-32. The concentration continues to increase in the third trimester, allowing fetal antibody concentrations to exceed maternal levels by 20-30%^9^. Studies suggest that the detection of immunoglobulin M in umbilical cord blood is more common in women who are vaccinated in the second or third trimester of pregnancy as already evidenced in studies of the vaccines for tetanus and influenza^8^ ^,^ ^9^ ^,^ ^10^.

Therefore, immunization during pregnancy can increase maternal immune protection and provoke the production and transfer of antibodies across the placenta to provide early infant protection. Recent studies have shown that this strategy is a safe and efficient means for protecting mothers and infants from vaccine-preventable infections^11^. 

Vaccination during pregnancy is a strategy to improve the health of mothers, fetuses, and newborns. Understanding the characteristics of antibody transfer is crucial to the development of a vaccine to help protect newborns^12^. However, it is important to conduct longitudinal studies on the duration of immunity for both mothers and children as well as the need for a third dose or vaccine booster. Children under 12 years of age are currently not covered by the National Immunization Plan against COVID-19 in Brazil. 

In this case report, passive immunity to the fetus was observed through maternal vaccination. The mother was exposed to inactive virus, a technology used in the CoronaVac® immunizer. The two doses administered at 31 and 34 weeks of gestation provided an increase in the mother’s total antibodies and, consequently, provided immunity to the fetus as assessed by the total antibody response.

To the best of our knowledge, this is the first case of newborn immunization after vaccination of the mother during the third trimester of pregnancy with the CoronaVac® vaccine. The monitoring of this and similar cases is essential to estimating the duration of circulating antibodies in the child and the role of breastfeeding in maintaining immunity against SARS-CoV-2.
